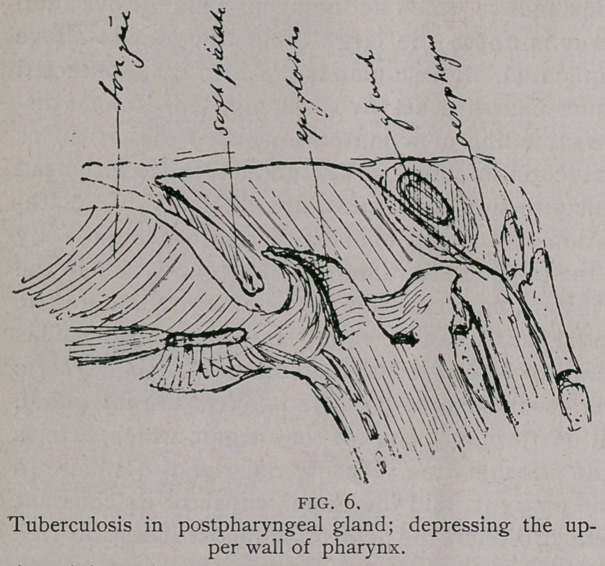# Actinomycosis

**Published:** 1892-03

**Authors:** R. W. Hickman, Victor A. Norgaard

**Affiliations:** Chief Inspector, Bureau of Animal Industry, at Chicago; Graduate of the Royal Veterinary and Agricultural College, Copenhagen, Denmark


### ACTINOMYCOSIS.

By Dr. R. W. Hickman, Chief Inspector, Bureau of Animal Industry, at Chicago, and Dr. Victor A. Norgaard, Graduate of the Royal Veterinary and Agricultural College, Copenhagen, Denmark.

For a considerable length of time previous to the discovery of the true cause of Actinomycosis, it was observed that cattle were afflicted with some disease affecting the bones and soft tissues of the head and throat, which received various popular names by farmers and dairy people, whose cattle were victims to it. It is only of late years that the medical professions have been able to throw much light upon the subject. The diagnosis is comparatively easy in cattle if external local lesions are present; and cases are exceedingly rare, as far as ascertained, either in this country or Europe, where the lesions of actinomycosis were first discovered in the viscera with no external evidence of the disease. The exceptions, however, in some cases where diagnosis is important, will be noticed farther on. The tumor will present itself in the lower jaw, sub-maxillary space, parotid and pharyngeal regions, super maxillary, tongue or face, in frequency in about the order mentioned, its contour depending largely upon the character of the tissue involved and the distance from the surface of the point primarily affected.

FIG. I.

Most frequent appearance.

There are no noticeable constitutional symptoms except those resulting from interference with the nominal performance of function by organs or parts affected. It has been claimed that the metastatic process is entirely through continuity or contiguity of tissue by some and by others that it occurs through the lymphatics. Be that as it may, while actinomycosis unquestionably does give metastasis, there is certainly a decided tendency to localization. Only yesterday we secured a large actinomycotic tumor from the loose connective tissue on a line paralel with the inferior border of the lower maxilla, just beneath the parotid gland, and none of the adjacent glands were affected.

This disease was described by Peroncito and Rivolto in 1869, and by Hahn in 1870, but Bollinger first recognized it as being of parasitic origin in 1877, and described the micro-organism or ray fungus which is its specific cause, may be invariably found, and is plainly diagnostic. The actinomyces with their specific effects are most frequently met with in the animal organism in jaws of cattle and in the milk glands of swine, though there is scarcely any organ or tissue that can be regarded as secure against the invasions of this parasite, if the circumstances are favorable to its entrance, into the organism of these or other animals, including man. In Denmark, actinomycosis was first described by Stockfleth, but as he failed to recognize its parasitic origin, he designated it as a chronic inflammation of the salivary glands. As his description, however, coincides with the symptoms and appearances now recognized, it is very evident that he was dealing with the same disease.

Microscopically, the fungus appears as small, round, whitish or straw yellow colonies about the size of a grain of sand, which can readily be seen in the pus drops squeezed out of the actinomycotic granulomas. The favorite seats of this disease, it may be said, are the jaws, the salivary glands, the tongue and post pharyngeal lymphatic glands. The parasite gains access to the animal organism most frequently through the alimentary canal and lives in nature on plants. Brazzola claims that the fungus lives especially on Hordeum Marinum; thus he showed the actinomyces between the fibres of small pieces of this barley lodged at the margin of the gums and between the teeth of cattle. If an abrasion or favorable condition is offered for its reception, an irritation results to the part, which is soon followed by a local inflammation, and under favorable circumstances the actinomyces will now multiply rapidly and spread to the surrounding tissue, and a tumor will appear which may be soft, of a sarcomatous consistance of a yellowish-red color, or it may be hard, of a grayish-white color and of a fibromatous nature or quite spongy, depending upon the character of tissue affected.

The tumor on the jaw usually consists of a fibrous stroma, in which will be found the small yellowish-red granulomas which may differ considerably in size. By making a section through such a tumor there will be seen a very characteristic picture. Up over the solid, white, fibrous tissue the granulomas will protrude conspicuously, which shows that they have been under pressure, out of these soft parts, by slightly squeeziug, small drops of the yellow, slimy pus will exude, containing the characteristic greenish-yellow grains. If the parasite follows the tooth or the inner wall of the alveola, gaining entrance to the jaw bone, the same process ensues, causing the bone to swell and undergo similar changes to those which occur in the softer parts; the teeth will be raised in the alveola, get loose or be eventually pushed out, the animal will be unable to masticate food, and of course the natural consequences will follow. This is one of the most quickly disastrous results of the disease to the individual. So if by location, the organs of prehension, mastication and deglutition are not rendered ineffective by the growth, the tumor may attain enormous size without interfering with the general health of the animal.

FIG. 2.

Aciinomycoses in the lower lip of a cow.

A most characteristic diagnostic appearance of actinomycosis is the formation of the granulomas on the surface, either on the cheek (after desquamation has occurred) or on the mucous membrane of the mouth, invariably distinctly seen in the latter. Should the germ reach the tissue beneath the mucus membrane of the mouth, possibly through some small accidental abrasion, there will result a small tumor which will show a strong tendency to suppuration, but in a very peculiar way, not simulating the same process in any other tumor formation, starting from a point in the middle where the supply of the necessary pabulum is least, but there will be many different foci in which pus formation will occur simultaneously, forming a large number of minute abcesses, which after a while will coalese and form a larger one containing constantly laudable and not putrefactive pus. This abcess will always try to empty itself out through the nearest surface, in this case into the mouth ; this done the process will not therefore come to an end by the cavity filling up with granulations and the opening healing up with a cicatrix, on the contrary the evacuation of the pus is only an encouragement to the actinomyces left adhering to the abcess wall, and by their influence the cavity will soon again fill up with the same soft yellowish-red granular tumor mass, and when the cavity is full, it will, like a fungus, grow out of the opening which has shown no tendency to healing. We will then have a mushroom form with its root in the cavity, the stem reaching through the opening, which, with the swelling of the surrounding parts, gives the tumor a very characteristic kind of drawn in appearance. These tumors show, although denied by many, a tendency to spontaneous healing. This can be explained by the shrinking of the surrounding fibrous tissue, which both compresses the base and stem and prevents a sufficient supply of nutrition. It has been observed in some cases that a large tumor has completely withdrawn through the opening, leaving nothing but a small depressed, contracted mark.

FIG. 3.

Actinomycosis developing.

Actinomycosis in the tongue is less frequently met with than in the maxilla. A German (Claus) claims to have seen twenty-nine per cent. out of a hundred and five cases in which the tongue was the primary seat of infection, fifty-one per cent. was in the maxilla and the balance in different locations. Another authority, Imminger, gives four to eight per cent. out of a hundred cases in which the tongue was the affected organ, while in from eighty-five to ninety per cent. the jaws were affected. The lesion in the tongue is usually designated "wooden tongue," (the German name "Holzzunge") and was previously regarded as tuberculous glossitis, as the disease runs a chronic course and commences with small tubercular-like nodes varying in size from that of a mustard seed to a pea. The first symptom usually manifested by the animal is slobbering during mastication, and refusing to eat the usual amount of food. It will be noticed now, by an examination of the mouth, that the tongue is sore and swoolen, and covered more or less with these small nodules. Some will be recognized as being under the mucus membrane, while others seem to be penetrating through it. The mobility of the tongue is, of course, considerably impaired, the sublingual glands are usually swollen, but not necessarily so. It may now easily be conjectured what the termination of the disease will be. In a few months at the longest, the tongue will be so hard and swoolen that the animal will be unable to close its mouth or to retain the tongue within the buccal cavity, or even to take fluid food, and consequently dies of starvation. There have been seen, however, cases of spontaneous recovery, but the tongue will never recover its original mobility nor shape. Through the shrinking of the fibrous connective tissue surrounding

FIG. 4.

Actinomycosis centre.

the tumors the organ will sometimes acquire strange forms, besides from six months to a year will be required for this abortive attempt at resolution. An instance in which actinomycosis may prove fatal in rather an acute way is where the large lymphatic glands above the pharynx are implicated. These glands are also found affected by tuberculosis, which causes them to swell, and the animal diseased to display the same line of symptoms which is described as occurring in actinomycotic infection. The animal stands with head extended on neck, manifesting uneasiness, has difficulty in breathing, and the respiration is accompanied by a roaring sound. By manipulation from the outside the tumor may be found, and if the fingers are pressed inward on both sides of the pharynx and a little over and before the larynx, it can be rather easily outlined. This tumor is pretty certain to be either of tuberculous or actinomycotic origin. In the latter case when the parasite has reached the gland, a small abscess will be formed inside of that organ, which, after a while will produce an inflammation, causing the gland to adhere to the upper wall of the pharynx, and the tumor will show its constant characteristic tendency to reach the nearest and most accessible free surface, which in this case will be into the pharynx, into which organ it will protrude as a polypus with a wide base. Now, by examination of the mouth it can be felt just behind the edge of the soft palate and immediately over the entrance to the larynx, and will always cause difficulty in breathing. The points by which a tuberculous affection of the pharyangeal glands may be differentiated from the actinomycotic, with care may be recognized. In the former affection the process will usually be strictly confined to the gland itself, resulting in a profuse swelling of that organ, which may attain enormous size ; and there will result the formation on of an abcess in the center, which abcess will be surrounded by a thick fibrous capsule, and the pus contained therein will be of a course caseus nature, often containing lime salts. The

FIG. 5.

Actinomycoses in postpharyngeal gland with tumor protruding into the pharynx. Traverse section through pharynx and larynx.

tuberculous lymphatic gland restricts the respiration by depressing the upper wall of the pharynx. By inserting the hand, fluctuation may occasionally be felt, as once in a while the abcess will open into the pharynx, but out of the opening through which the pus has been evacuated we will *never* have the granulomatous growth. And with this difference in mind, it will be comparatively easy to distinguish between the two diseases, which may be of very great importance both to the owner and practitioner.

FIG. 6.

Tuberculosis in postpharyngeal gland; depressing the upper wall of pharynx.

Actinomycocis in the salivary glands is quite a frequent occurrence. The majority of these cases are seen in the parotid. They are supposed to extend to this gland from the underlying lymphatic glands, but the germs possibly reach the salivary glands by means of the ducts or natural openings, through a similar process by which they gain access to the mamary glands. The tumors are rather hard, vary somewhat in size, and can for a long while remain unchanged, but at last will open in one or more places, and through these openings, after the evacuation of the pus, there will grow the characteristic soft granulomas. The cause of a great number of cases affecting localities within and around the mouth and pharynx has already been mentioned, but animals may also be infected by means of the inhaled air, resulting in disease of the lungs and other parts of the respiratory apparatus.

Prof. Bang, of the Royal Veterinary and Agricultural College of Copenhagen, Denmark, described one case of miliary actinomycosis of the lungs The most plausible explanation is that the micro-organisms were carried by the plasma current, or transported by the leucocytes. This condition, however, is excessively rare. Some also have nodes of different sizes, more or less numerous. They all, however, have the characteristic granulous structure, and are usually confined to a certain part of the lung. For instance, one-half of one lung may have a hard indurated appearance and by cutting this part through we will find the lung tissue transformed into a fibrous tendon-like mass in which the granulous will appear with more or less space between them, some of them intersecting and others running into each other and forming larger ones. In a single case there has been found in such a fibrous part of a lung one enormous actinomycotic tumor about the size of the head of a child. In older cases the pleura pulmonalis may be implicated, and in some instances the lesion involves the pleura costalis, the pleural surfaces having become adherent, they will assume a swollen dense appearance, and on the pleura costalis may be seen neoplasms of connective tissue and stalky actinomycomas, which may penetrate into and involve the intercostal muscles, and will no doubt, if the animal lives sufficiently long, give the same picture of disease as in man, where it comes to the formation of fiistulas out through the skin. In two cases reported from the stockyards in Copenhagen the actinomycotic lesions had gone from the lungs over into the pleura costalis, and from there without respecting the difference in character of the tissue, in one case attacked the fifth rib, and in both cases gone through the intercostal muscles so that numerous actinomyces were seen on the outside of the chest after the carcass had been skinned.

The lymphatic glands around the entrance to the chest cavity are usually affected in the more exaggerated forms of the disease. To distinguish actinomycosis from tuberculosis in the lungs, of a live animal, is almost impossible without obtaining the sputum and recourse to the microscope ; but in nearly all casns of actinomycosis in the lungs, we will find the disease somewhere else ; for instance, around the teeth, on the jaws, or in the tongue, and can thus make a diagnosis from those. Professor Bang has also seen both diseases, actinomycosis and tuberculosis, in the same lung.

Of the organs in the abdominal cavity, the liver is most liable to be attacked by actinomycosis. At the stockyards in Copenhagen in 1890, the disease was proven to exist in twenty-two ox livers. In 1391 Veterinary Inspector P. B. Rasmussen reports that on the 16th of January he saw killed an old cow, which exhibited no symptoms of the disease while alive. At the post-mortem examination the right lung was found adherent to the diaphragm, and contained an actinomycotic tumor as big as a clinched fist ; the right side of the diaphragmatic abdominal surface was covered with fringes and connective tissue, and from the anterior surface of the right side of the liver, near its upper margin, protruded an actinomycotic tumor about the size of a head, covered with a fibrous capsule. To this capsule the anterior end of the right kidney was adherent, and on the surface of both kidneys, on the right four and on the left thirteen, white, round actinomas, varying in size from that of a pea to a chestnut, were prominently exhibited. And beside those already mentioned there was one more actinomycoma in the liver about the size of a goose egg.

The same authority quotes a case of actinomycosis in the large lymphatic glands in the posterior parts. The animal was a big fat steer which showed no symptoms of the disease while alive. At the post-mortem examination on the left inguinal glands were found swollen to about the size of the double clinched fists, and the left sublumbar glands as large as a single fist. The lesions proved to be of actinomycotic nature. The pharyangeal glands were also involved and full of actinomycotic deposits. In the lungs nothing was found, and as the intestines had been thrown away by the butcher nothing can be said of them. This case would also appear to prove that the disease may decimated by the blood or plasma current.

Actinomycosis in the udder of a milch cow was first described by Johme, who succeeding in infecting the udder of a cow artificially. Several cases of actinomycosis of the udder, however, have been seen at the stockyards in Copenhagen. The disease may present different appearances, the udder may be greatly swollen and hard, or possibly loose and flabby. By manipulation of the affected part nodes and tumors of every size, from that of a pea to a hen's egg, may be felt. They are hard and uneven, sometimes even angular. The nodes first appear at the base of the teat and proceed upwards ; they are frequently arranged in rows. The large lymphatic glands at the base of the udder may be hard and swollen, but not necessarily so. The disease may make very rapid progress, which may soon lead to the total destruction of the affected gland, or it may come to a termination by nature's attempt at recovery through calcification, without greater changes in its tissue. It has not been proved that the milk is infected, but it is safest to assume that it is, as the tumors may be situated everywhere in the gland tissue. Inspector Rasmussen describes four or five cases, seen by himself, in which two were of a violent form in milking cows, while the others were of a more good-natured sort, and were found in dry cows. In none of these cases were there any symptoms of acute inflammation, neither was their evidence of the disease in any other organ or tissue of the body, consequently the parasite gained access to the diseased gland through the natural opening. By making a section through such an udder it will show the affected part indurated, the gland tissue converted into fibrous tissue, and the granulomas of different sizes imbedded in this. The actinomyces grains are usually very large and contain plenty of lime salts, which has to be dissolved in acetic acid before microscopic examination. Actinomycosis in the milk glands of the sow is very common. The gland tissue becomes fibrous and tendon-like, and the actinomycomas will be seen protruding through the surface, either singly or in a circle round the teat, or are more deeply situated, and may, by manipulation, be felt as small, round, hard nodes, varying in size from that of a pea to a cherry, seldom larger. It is only once in a while that they show a tendency to penetrate the skin. By cutting through a section we see the alveola sometimes surrounded by a red zone and containg a caseous mass, in which the yellow actinomyces appear conspicuously.

Inoculation may occur through a mere accidental wound in any part of the body. I will illustrate by describing a couple of samples from the stockyards at Copenhagen.

A fat calf four or five months old had on the middle of its back, slightly to the left of the spinal column, two large red, round sarcomatous tumors, both about the size of an egg ; one a little bigger than the other. The neoplasms protruded through the skin in one direction, and through the muscles of the back down into the upper end of the underlying rib on the other. The periostium on the rib was swollen and tense, and the rib itself was increased considerably in circumference, so that it extended over the margins of the adjacent ribs on the pleural surface. A section from the tumor gave the characteristic picture of the actinomycoma, and the presence of the parasite was easily proven by microscopic examination in the pus squeezed out of the soft parts, and nowhere else were found any actinomycotic lesions. A cow killed at the same place had on the outside of the right shank a large swelling, which was supposed to be an abscess, but proved, after butchering, to consist of several actinomycomas. There was no evidence of actinomycosis elsewhere in the body.

Actinomycosis gain access to the animal organism through sores from operations, such as castration and spaying. Instance, a four-year old steer, in good condition, was killed in June, 1890. There was considerable induration of both cords. The swelling in connection with the surrounding adipose tissue formed a tumor about the same size as the natural scrotum of a bull of the same age. At the post-mortem was found on the end of the right cord a well defined tumor of the form of a pear and about the size of a goose egg. Upon incision it proved to be an abscess filled with knotted yellowish-gray pus, in which were a number of small yellow lime grains; the abscess wall had the characteristic actinomycotic structure, on the outside fibrous masses surrounding the soft grayish granulomas, out of which pus drops could be squeezed in which actinomyces were recognized. The tumor in the left cord was somewhat larger and broader in the lower end, and not as well defined from the surrounding tissue. It contained in the lower end, however, a typical actinomycoma of the size of a hen's egg, and a little above this a couple of small actinomycomas about the size of a pea. The parasite was proven to be present in all of them.

While many cases of scirrhous cord have been attributed to infection from actinomyces, it is highly probable that in the majority of these cases the lesions were the direct result of an entirely different micro organism, viz: micrococcus botryogenes; also called micrococcus ascoformans, or botryococcus ascoformans; it is only mentioned here because the lesions produced by this parasite may very closely resemble those resulting from actinomyces, in that we may have the same fibrous formations surrounding softer parts and caverns, and the pus therein contained will exhibit the same small yellowish granules; by examining these, under the microscope, a difference will be observed with a low magnification, as in the botryogenes ascoformans we will have a conglomeration of small, round bodies of different sizes arranged together somewhat in the shape of a blackberry or a minute cluster of grapes, hence the name Ascoformans. By pressing one of these granules between two covered glasses, then staining and using a stronger magnification, you will see that the grape-shaped bodies have been transformed into numbers of micrococci. A German authority, Kitt, claims that it is only a variation of staphylococcus pyogenes aureus; this lesion may also be found in the lungs as metastassi, and also here resembles actinomycosis very much, but a microscopial examination will soon make the diagnosis sure.

## Figures and Tables

**FIG. 1. f1:**
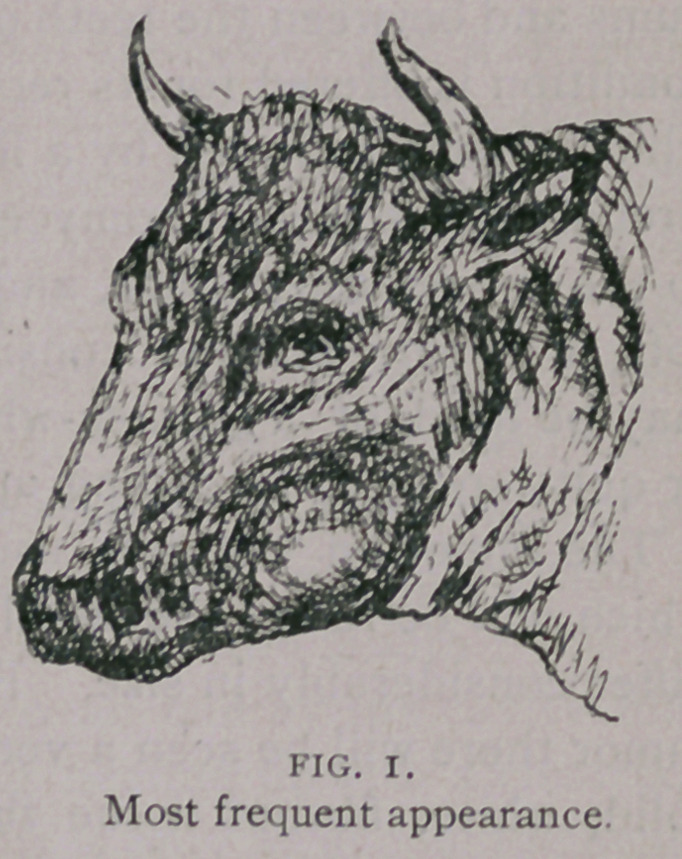


**FIG. 2. f2:**
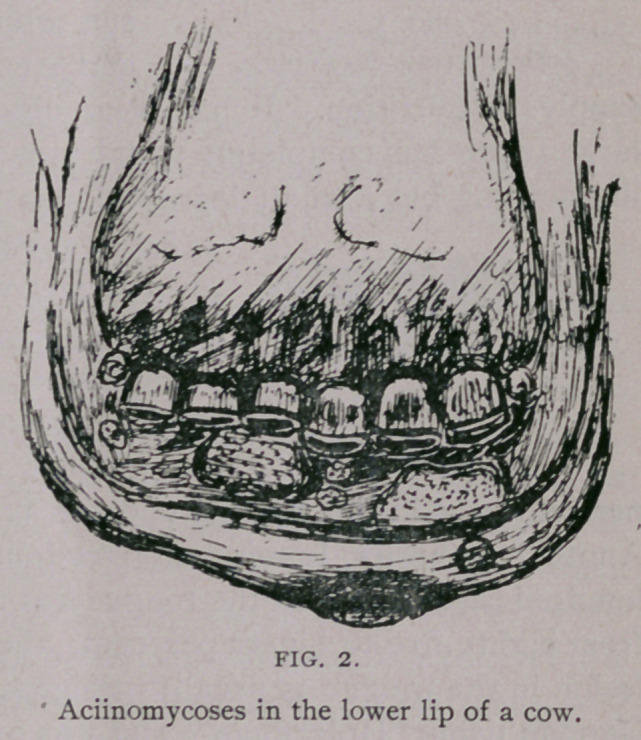


**FIG. 3. f3:**
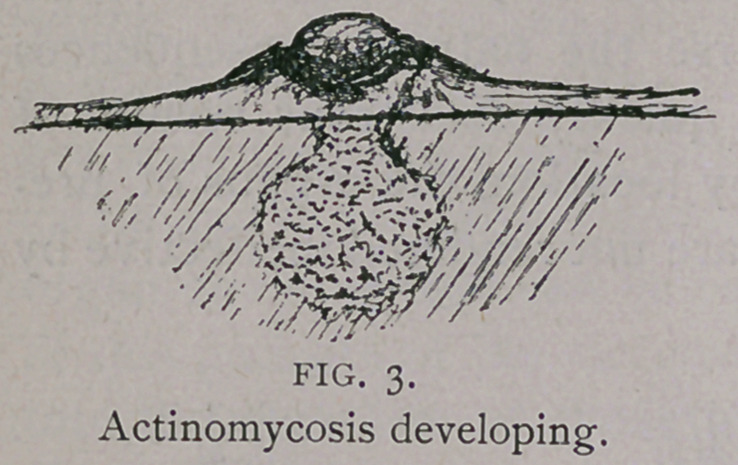


**FIG. 4. f4:**
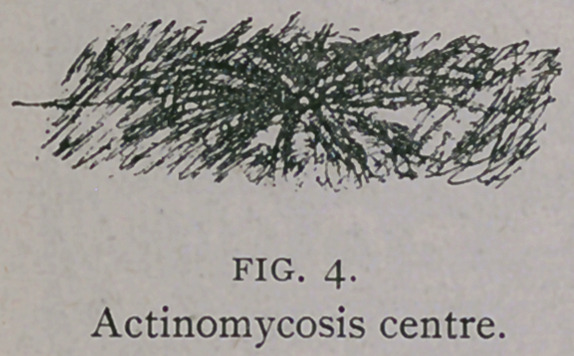


**FIG. 5. f5:**
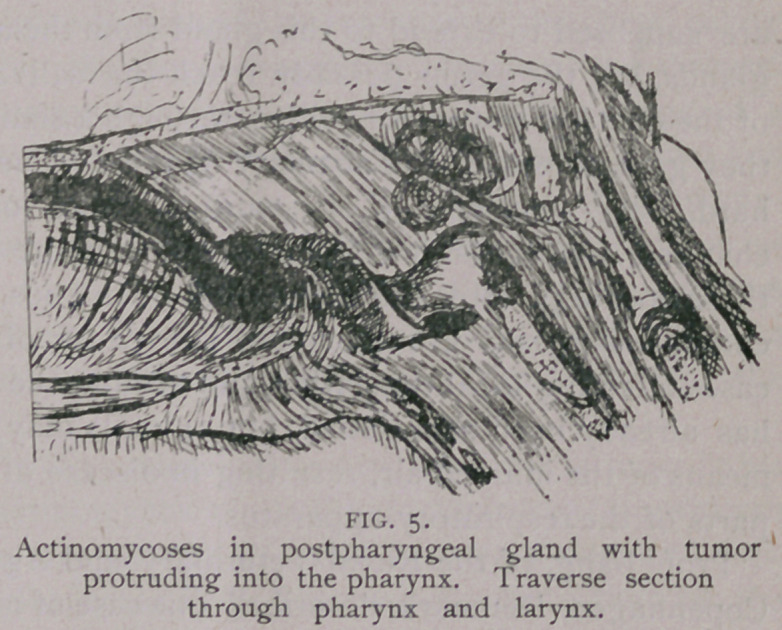


**FIG. 6. f6:**